# Mycobacterial OtsA Structures Unveil Substrate Preference Mechanism and Allosteric Regulation by 2-Oxoglutarate and 2-Phosphoglycerate

**DOI:** 10.1128/mBio.02272-19

**Published:** 2019-11-26

**Authors:** Vítor Mendes, Marta Acebrón-García-de-Eulate, Nupur Verma, Michal Blaszczyk, Márcio V. B. Dias, Tom L. Blundell

**Affiliations:** a Department of Biochemistry, University of Cambridge, Cambridge, United Kingdom; b Department of Microbiology, Institute of Biomedical Science, University of São Paulo, São Paulo, Brazil; c Department of Chemistry, University of Warwick, Coventry, United Kingdom; Weill Cornell Medical College

**Keywords:** *Mycobacterium*, OtsA, trehalose-6-phosphate synthase, trehalose

## Abstract

Trehalose is an essential disaccharide for mycobacteria and a key constituent of several cell wall glycolipids with fundamental roles in pathogenesis. Mycobacteria possess two pathways for trehalose biosynthesis. However, only the OtsAB pathway was found to be essential in Mycobacterium tuberculosis, with marked growth and virulence defects of OtsA mutants and strict essentiality of OtsB2. Here, we report the first mycobacterial OtsA structures from Mycobacterium thermoresistibile in both apo and ligand-bound forms. Structural information reveals three key residues in the mechanism of substrate preference that were further confirmed by site-directed mutagenesis. Additionally, we identify 2-oxoglutarate and 2-phosphoglycerate as allosteric regulators of OtsA. The structural analysis in this work strongly contributed to define the mechanisms for feedback inhibition, show different conformational states of the enzyme, and map a new allosteric site.

## Abstract (precis)

Mycobacterial infections are a significant source of mortality worldwide, causing millions of deaths annually. Trehalose is a multipurpose disaccharide that plays a fundamental structural role in these organisms as a component of mycolic acids, a molecular hallmark of the cell envelope of mycobacteria. Here, we describe the first mycobacterial OtsA structures. We show mechanisms of substrate preference and show that OtsA is regulated allosterically by 2-oxoglutarate and 2-phosphoglycerate at an interfacial site. These results identify a new allosteric site and provide insight on the regulation of trehalose synthesis through the OtsAB pathway in mycobacteria.

## INTRODUCTION

Trehalose is a nonreducing disaccharide, formed by α-(1-1)-linked glucoses, with a wide distribution in nature and present in all three domains of life (1). This remarkably widespread sugar performs multiple roles in a wide variety of organisms, and it can also fulfill different roles within the same organism. Trehalose has been considered a compatible solute, conferring protection on proteins, DNA, membranes, and whole cells from thermal shock, osmotic shock, freezing, ionizing radiation, oxidative stress, and desiccation (1–9). This disaccharide, which can further function as a carbon and energy reserve molecule (1, 10–12), was also recently related to the pathogenicity of Pseudomonas aeruginosa in plants (13). It additionally plays fundamental signaling roles in plants, in the phosphorylated form (trehalose-6-phosphate), where it regulates sucrose metabolism and flowering (1, 14–16), and in yeast, where it regulates gluconeogenesis and glycolysis (17, 18). Trehalose was further shown to be an autophagy inducer, both in plants and in mammals, with potential biotechnological and clinical implications (19, 20).

In mycobacteria, trehalose is also an essential component of mycolic acids and other cell wall glycolipids, which are major protagonists in pathogenesis of Mycobacterium tuberculosis (1), the causative agent of the widespread infectious disease tuberculosis. In these organisms, trehalose was further identified as a key signaling molecule of cell envelope stress, playing a role as an activator of the *iniBAC* operon, which is induced when mycobacteria are exposed to the first-line drug isoniazid (21).

All mycobacterial species, with a few exceptions, possess two pathways to synthesize trehalose, the OtsAB and the TreYZ pathways (1). The OtsAB pathway is the most widely distributed trehalose biosynthesis pathway, present in bacteria, archaea, and eukaryotes (22, 23). In this pathway, which is conserved and essential in mycobacteria, trehalose is synthesized in a two-step process involving OtsA and OtsB2 enzymes. M. tuberculosis mutants showed that the OtsAB pathway was not only the dominant route for trehalose biosynthesis in this pathogen but also required for growth both *in vitro* and in a mouse infection model, with marked growth and virulence defects of OtsA mutants (24) and strict essentiality of OtsB2 due to the toxic effect of trehalose-6-phosphate (T6P) accumulation (24, 25).

OtsA, a glycosyltransferase that belongs to the GT20 family of the CAZY classification (www.cazy.org), uses the α anomer of glucose-6-phosphate (G6P) as acceptor and nucleoside diphosphate glucose (NDP-glucose) as the donor to synthesize T6P, with net retention of the anomeric configuration of the donor substrate (26, 27). Binding of the substrates occurs sequentially in a “bi-bi mechanism” with NDP-glucose binding first and G6P second (26, 28). Interestingly, OtsAs from different organisms show different donor preferences as shown by kinetic studies and X-ray crystal structures, but the reasons behind these preferences are poorly understood (27, 29–33). Recently, different roles were identified for OtsA beyond its enzymatic activity. OtsA was reported to act as an osmotic stress sensor and morphogenetic protein that can regulate the switch to mycelioid growth in *Arthrobacter* sp. strain A3, a pleomorphic soil-dwelling actinobacterium (34).

In this work, we purified, crystallized, and solved the structure of Mycobacterium thermoresistibile OtsA (*Mtr*OtsA). To gain further insight into the substrate preference of mycobacterial OtsAs, we have obtained structures with substrates that define the mechanisms of ADP-glucose preference. Structure-guided point mutations of key residues in the active site were performed, and characterization of the mutants showed that three mutations are enough to change the donor substrate preference to UDP-glucose. Further structures with OtsA product and pathway product were obtained, showing how this enzyme is feedback inhibited by trehalose. Importantly, with these structures, we have also identified a new allosteric site and allosteric regulators of this enzyme that link glycolytic and tricarboxylic acid (TCA) cycle metabolites to the regulation of trehalose synthesis.

## RESULTS

### Overall structure.

**FIG 1:**
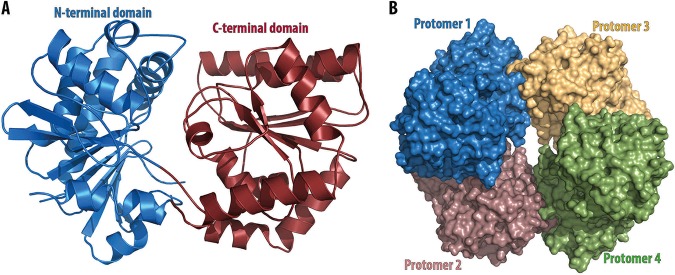
(A) Representation of the overall structure of *M. thermoresistibile* OtsA. The N-terminal domain consists of residues 1 to 247 and 462 to 486, and the C-terminal domain consists of residues 248 to 461. (B) View of *M. thermoresistibile* OtsA tetramer.

The structure of *Mtr*OtsA in its apo form was solved by molecular replacement, using the Escherichia coli OtsA structure (PDB code 1UQU) as the search model (see Fig. S1 in the supplemental material). Data collection and refinement statistics are summarized in Table S1. *Mtr*OtsA is composed of two Rossmann-fold domains with a deep catalytic site at their interface, in an arrangement typical of GT-B glycosyltransferases as described previously for other organisms (27, 29, 30). The apo protein crystallized in the I4_1_22 space group, with one protomer per asymmetric unit, and diffracted to ∼1.8-Å resolution. The N-terminal domain is formed by a core of 7 parallel β-strands flanked on both sides by an antiparallel β-strand and surrounded by 8 α-helices, one of which is composed of the final C-terminal residues (Fig. 1A). The C-terminal domain contains 6 parallel β-strands associated with 9 α-helices with the last one undergoing a kink and extending to the N-terminal domain, characteristic of the GT-B fold glycosyltransferases (Fig. 1A). Analysis of the B-factor distribution shows that the N-terminal domain has the highest values for atomic temperature factors, suggesting that this domain is more dynamic, which is consistent with the large movements of α-1 during catalytic activity.

10.1128/mBio.02272-19.1 FIG S1 Alignment of the X-ray crystal structures of E. coli OtsA (PDB code 1UQU) in cyan and *M. thermoresistibile* OtsA Apo form in orange (root mean square deviation [RMSD] = 2.996). Download FIG S1, DOCX file, 0.4 MB. Copyright © 2019 Mendes et al. 2019 Mendes et al. This content is distributed under the terms of the Creative Commons Attribution 4.0 International license.

10.1128/mBio.02272-19.8 TABLE S1 X-ray crystallography data collection and final refinement statistics. Download Table S1, DOCX file, 0.03 MB. Copyright © 2019 Mendes et al. 2019 Mendes et al. This content is distributed under the terms of the Creative Commons Attribution 4.0 International license.

*Mtr*OtsA forms a tetramer in solution, as previously observed for M. tuberculosis OtsA. This tetrameric form is also observed in all the crystal structures reported here (Fig. 1B). The amino acids involved in the tetramer interfaces are not conserved beyond mycobacteria and related species (Fig. S2), suggesting that OtsA might have a different molecular assembly in different species. Indeed, in E. coli both tetrameric and dimeric forms were reported (27) while in Streptomyces venezuelae and Aspergillus fumigatus only the dimeric form was observed (29, 30). However, in mycobacteria and closely related organisms the tetramer interfaces are highly conserved, suggesting a tetrameric assembly of OtsA in all of these organisms (Fig. S2).

10.1128/mBio.02272-19.2 FIG S2 Sequence comparison of OtsA from Mycobacterium thermoresistibile, Mycobacterium tuberculosis, Mycobacterium leprae, Mycobacterium avium, Mycobacterium abscessus, Nocardia farcinica, Arthrobacter alpinus, *Salmonella* Typhimurium, Escherichia coli, Candida albicans, Paraburkholderia xenovorans, and Streptomyces venezuelae. Residues that contact the substrates are highlighted with blue circles (donor site) and green circles (acceptor site). The allosteric site residues are marked with crosses, and tetramer interfaces are highlighted in red. The tetramer interfaces are highly conserved only in the several mycobacteria and *N. farcinica* and less conserved in *A. alpinus*. The remaining nonactinobacterial species show very little conservation of the interfaces. The same is observed for the allosteric site. Acceptor site residues are conserved throughout. Donor site residues are less conserved, indicating the known differences in substrate preference. Download FIG S2, DOCX file, 0.3 MB. Copyright © 2019 Mendes et al. 2019 Mendes et al. This content is distributed under the terms of the Creative Commons Attribution 4.0 International license.

### Catalytic site.

**FIG 2:**
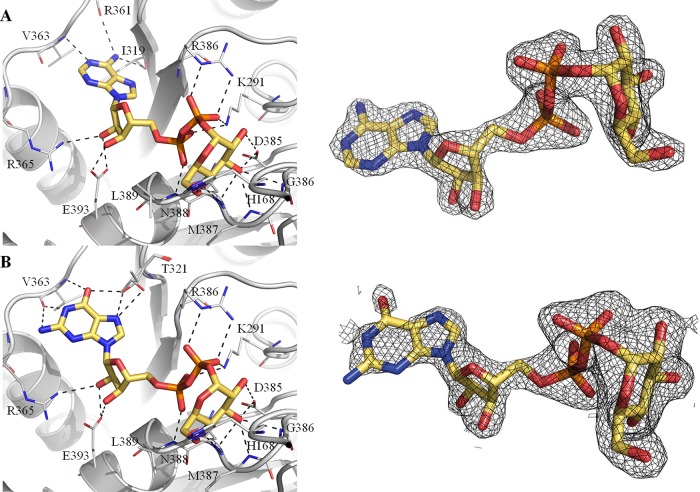
Detailed view of the active site of *M. thermoresistibile* OtsA with ADP-glucose (A) and with GDP-glucose (B) bound with Fo-Fc “omit maps” shown contoured at 1.5 σ. Black dashed lines represent hydrogen bonds.

**FIG 3:**
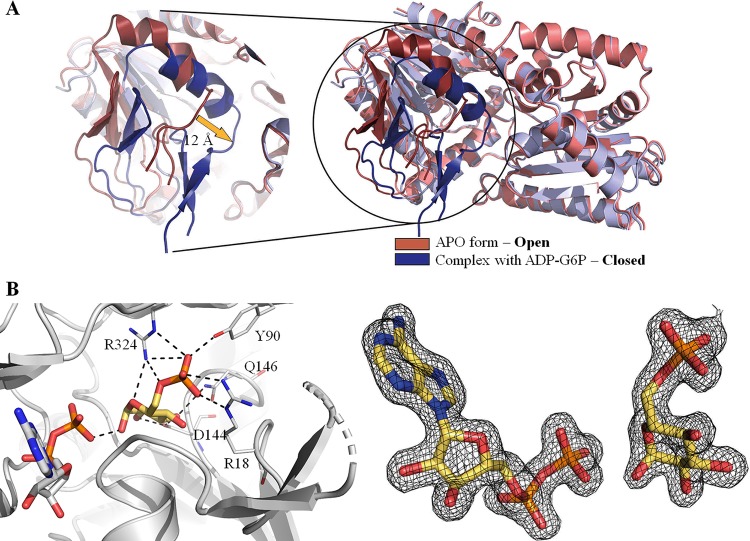
(A) View of OtsA in an open (salmon and red) and a closed (violet and blue) conformation and superposition of the two conformations. (B) Representation of the active site of *M. thermoresistibile* OtsA with ADP (white) and G6P (yellow) bound with Fo-Fc “omit map” shown contoured at 1.5 σ. Black dashed lines represent hydrogen bonds.

OtsA is a glycosyltransferase that uses the α anomer of G6P as acceptor and NDP-glucose as the donor to synthesize T6P. The *Mtr*OtsA catalytic site is located between the two Rossmann fold domains in a large and deep cavity. Structures with donor substrates were determined by soaking the apo form crystals with ADP-glucose and GDP-glucose (Fig. 2). The donor substrates interact primarily with the C-terminal domain through the side chains of the highly conserved Arg286, Lys291, Asp385, and Glu393 and with the side chain of Arg365 (Fig. 2), which is conserved in all mycobacteria and closely related species but less conserved outside this group (Fig. S2). Backbone interactions with the absolutely conserved residue Gly386, Met387, Asn388, and Leu389 amine groups are also observed. Similar interactions were observed before for E. coli OtsA (27, 32). While the adenine moiety interacts with the Val363 amino group, the Leu319 carbonyl, and a coordinated water (Fig. 2A), the guanine moiety forms interactions with Val363 only (Fig. 2B). N-terminal domain interactions with the donor substrate are observed only for His168 when the active site is in an open conformation (Fig. 2). To obtain a structure with the acceptor substrate, we cocrystallized OtsA in the presence of 5 mM ADP and G6P. As G6P binds to the protein in the presence of a donor substrate or its nucleotide, the active site adopts a closed conformation (Fig. 3A). The OtsA:ADP:G6P ternary complex crystallized in the P6_2_22 space group, with one protomer per asymmetric unit, and the crystals diffracted up to ∼1.7-Å resolution. In this closed conformation, new interactions with the donor substrate are formed with the Gly39 peptide-NH function (Fig. 3). Due to the absolute conserved nature of Gly39 among all functional OtsAs and related enzymes (27) and the contact that it forms with the glucose-bound phosphate, this residue is highly likely to be mechanistically involved in the catalytic activity (27).

The acceptor interactions recapitulate what was observed before for E. coli (27) with the substrate interacting with the highly conserved residues Arg18, Tyr90, Asp144, and Gln146 of the N-terminal domain and with only a single residue of the C-terminal domain, Arg324 (Fig. 3B). The residues involved in acceptor substrate interaction are highly conserved across both bacteria and yeast (Fig. S2). As described for E. coli OtsA (27), the OtsA:ADP:G6P ternary structure shows the catalytic site in a closed conformation that substantially differs from the apo form, with α-helix 1 and the Arg35-Gly39 loop region moving up to ∼12 Å (Fig. 3A).

### Properties of *M. thermoresistibile* OtsA.

*Mtr*OtsA uses ADP-glucose, UDP-glucose, and GDP-glucose as glucose donors with a preference for ADP-glucose (Table 1) and G6P as the only acceptor, in accordance with what was reported before for M. tuberculosis OtsA (33, 35). The kinetic parameters for *Mtr*OtsA were obtained and are reported in Table 1 and Fig. S3 with *K_m_*^app^ values for the preferred donor substrate (ADP-glucose) of 0.25 ± 0.02 mM and for the acceptor (G6P) of 3.3 ± 0.1 mM. The *K_m_*^app^ for GDP-glucose of 0.29 ± 0.02 mM was in the same range as the one obtained for ADP-glucose; however, the turnover was 5-fold lower (Table 1). For UDP-glucose, the enzyme showed an ∼7-fold-higher *K_m_*^app^ of 1.7 ± 0.1 mM than the one obtained for ADP-glucose (Table 1).

**TABLE 1:** Kinetic parameters of *M. thermoresistibile* OtsA

Genotype and substrate	*K_m_*^app^ (mM)	*k*_cat_^app^ (s^−1^)	*k*_cat_/*K_m_* (s^−1^ · mM^−1^)
Wild type			
ADP-glucose	0.25 ± 0.02	26 ± 1	104 ± 13
GDP-glucose	0.29 ± 0.02	5.1 ± 0.2	18 ± 2
UDP-glucose	1.7 ± 0.1	36 ± 1	21 ± 2
Glucose-6-phosphate	3.3 ± 0.1	26 ± 1	7.9 ± 0.5
L319I			
ADP-glucose	0.71 ± 0.18	8.4 ± 0.7	12 ± 2
GDP-glucose	0.35 ± 0.04	3.1 ± 0.1	8.9 ± 1.4
UDP-glucose	1.4 ± 0.1	8.9 ± 0.4	6.4 ± 0.7
Glucose-6-phosphate	3.3 ± 0.2	6.4 ± 0.2	1.9 ± 0.2
V363F			
ADP-glucose	0.60 ± 0.03	11 ± 1	18 ± 2
GDP-glucose	0.74 ± 0.08	14 ± 1	19 ± 3
UDP-glucose	0.68 ± 0.06	13 ± 1	19 ± 3
Glucose-6-phosphate	3.1 ± 0.2	19 ± 1	6.1 ± 0.7
L319I, E367L			
ADP-glucose	0.63 ± 0.04	14 ± 1	22 ± 3
GDP-glucose	0.70 ± 0.05	9.2 ± 0.4	13 ± 2
UDP-glucose	0.63 ± 0.03	15 ± 1	24 ± 3
Glucose-6-phosphate	3.7 ± 0.2	21 ± 1	5.7 ± 0.5
L319I, V363F, E367L			
ADP-glucose	1.3 ± 0.1	19 ± 1	15 ± 2
GDP-glucose	0.90 ± 0.06	16 ± 1	18 ± 2
UDP-glucose	0.52 ± 0.05	22 ± 1	42 ± 6
Glucose-6-phosphate	3.2 ± 0.2	17 ± 1	5.3 ± 0.6
R213E			
ADP-glucose	0.31 ± 0.01	56 ± 2	180 ± 13
Glucose-6-phosphate	3.3 ± 0.3	33 ± 2	10 ± 1
R384E			
ADP-glucose	1.9 ± 0.2	3.2 ± 0.1	1.7 ± 0.2
Glucose-6-phosphate	2.9 ± 0.2	2.1 ± 0.2	0.77 ± 0.05

10.1128/mBio.02272-19.3 FIG S3 *Mtr*OtsA wild-type and mutant kinetics. G6P concentration was fixed at 10 mM for all NDP-glucose. ADP-glucose concentration was kept at 2.5 mM for G6P. Download FIG S3, DOCX file, 0.3 MB. Copyright © 2019 Mendes et al. 2019 Mendes et al. This content is distributed under the terms of the Creative Commons Attribution 4.0 International license.

The preference of ADP-glucose over the other glucose donors was further confirmed by isothermal titration calorimetry (ITC). The binding affinity of substrate donors could be determined only for ADP-glucose under the tested conditions with an observable *K_d_* (dissociation constant) of 27.17 ± 2.66 μM (Fig. S4). Other glucose donors analyzed (GDP-glucose and UDP-glucose) had no observable heat of binding, and the same was observed for G6P. The lack of heat of binding for GDP- and UDP-glucose is most likely due to their reduced affinity. In the case of G6P, it reflects a necessity of previous binding of the donor substrate.

10.1128/mBio.02272-19.4 FIG S4 ITC trace with *Mtr*OtsA for ADP-glucose binding. Download FIG S4, DOCX file, 0.08 MB. Copyright © 2019 Mendes et al. 2019 Mendes et al. This content is distributed under the terms of the Creative Commons Attribution 4.0 International license.

### Donor substrate preference is largely mediated by three residues.

**FIG 4:**
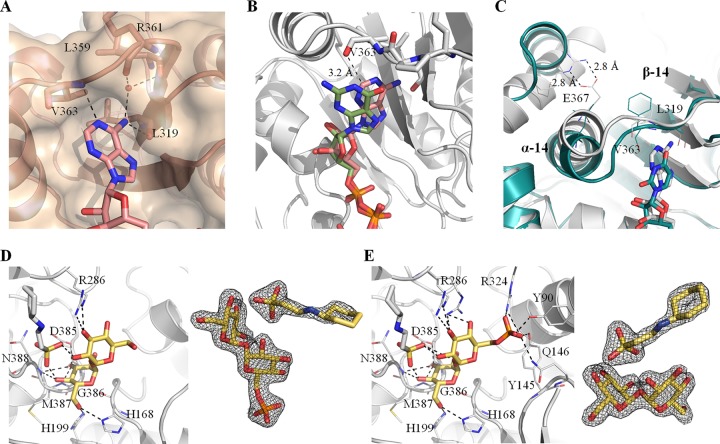
View of the binding site of the adenine moiety of ADP-glucose. (A) A water that mediates the adenine moiety interaction with OtsA is represented as a red sphere. (B) Superposition of ADP-glucose and GDP-glucose structures. (C) Superposition of *Mtr*OtsA structure with ADP-glucose bound and E. coli OtsA structure with UDP-glucose bound (PDB code 1UQU). α-helix 14 (α-14) and β-strand 14 (β-14) are shown. (D and E) Detailed view of the active site of *M. thermoresistibile* OtsA with trehalose (D) and with T6P bound (E) with CHES visible in both structures. Fo-Fc “omit maps” are shown contoured at 1.5 σ for trehalose, T6P, and CHES. Black dashed lines represent hydrogen bonds.

OtsAs from different organisms have been shown to have different donor substrate preferences, while being capable of using several nucleotide donors (30, 33, 36, 37), which is reflected in lower conservation of the donor substrate-interacting residues (Fig. S2). Preference for ADP-glucose as the donor substrate in *Mtr*OtsA is conferred by interactions with the deeply buried adenine moiety (Fig. 4A). The carbonyl groups of Leu319 and Arg361 interact with the primary amine, and the amide group of Val363 interacts with N-1 of the adenine moiety (Fig. 4A). A highly coordinated water interacts with the primary amine of the adenine moiety and the carbonyl groups of Leu359 and Arg361. The guanine moiety of GDP-glucose does not occupy the same deeply buried pocket as the adenine because its primary amine group would sterically clash with the Val363 carbonyl group (Fig. 4B). It thus binds to OtsA more weakly than ADP-glucose, explaining the substrate preference and consequently the lack of observable heat of binding under the tested ITC conditions. A binary complex structure with OtsA and UDP-glucose was also obtained, but the electron density was observed for only glucose and the two phosphates (not shown), indicating a reduced preference for this substrate as confirmed by enzymatic and biophysical data. Comparing the OtsA structures of *M. thermoresistibile* and E. coli, we hypothesized that the ADP preference was mediated by the substitution of an isoleucine (Ile295 in E. coli) for a leucine (Leu319 in *M. thermoresistibile*). The presence of the leucine in *M. thermoresistibile* allows the primary amine of the adenine moiety to occupy a buried position, interacting with the carbonyl group of Leu319 (Fig. 4C) which no other nucleotide activated donor can occupy. However, given the differences between the two enzymes, it is likely that other residues could also play a role. To improve the selection of residues to mutate, we employed a computational approach using mCSM-lig (38), a software program developed by our group that predicts the effects of mutations on binding affinity. The software predicted that several residues within 4.5 Å of the adenine moiety have a destabilizing effect on the interactions between *M. thermoresistibile* OtsA and ADP-glucose if mutated to the E. coli OtsA equivalent (Table S2). Combining this information with structural analysis, three mutations predicted to be destabilizing were selected. These mutations are Val363Phe, Leu319Ile, and Glu367Leu, the last being a long-distance mutation that we hypothesized would have a strong effect on ligand binding.

10.1128/mBio.02272-19.9 TABLE S2 mCSM-lig predictions. Download Table S2, DOCX file, 0.01 MB. Copyright © 2019 Mendes et al. 2019 Mendes et al. This content is distributed under the terms of the Creative Commons Attribution 4.0 International license.

To confirm our hypothesis, we generated several *Mtr*OtsA mutants (Leu319Ile, Val363Phe, Leu319Ile-Glu367Leu, and a triple mutant) and obtained kinetic data for these mutants (Table 1). As predicted, the mutant Leu319Ile showed an ∼3-fold increase in the *K_m_*^app^ for ADP-glucose whereas *K_m_*^app^ values were similar for GDP-glucose and UDP-glucose compared to the wild type. Even though GDP-glucose now showed the best *K_m_*^app^ among the three different donors, the catalytic efficiency and turnover rate were still higher for ADP-glucose (Table 1). A complete reversal of the donor substrate preference for UDP-glucose was obtained only with a combination of the three mutations (Table 1). The triple mutant showed an ∼5-fold increase in ADP-glucose *K_m_*^app^ and a reduction for the UDP-glucose *K_m_*^app^ by more than 3-fold (Table 1). The catalytic efficiency was also completely reversed for ADP- and UDP-glucose between the wild type and the triple mutant (Table 1). Although Leu319Ile showed the largest contribution to ADP-glucose *K_m_*^app^ increase, Val363Phe and Glu367Leu were also determinant for the reversal to UDP-glucose preference (Table 1). This can be explained by the fact that the phenylalanine substitution at position 363 forces the loop between β-14 and α-14 to move toward the nucleotide binding site (Fig. 4C), thus establishing stronger interactions with a pyrimidine nucleotide and clashing with purine nucleotides. This effect is also observed for both ADP- and GDP-glucose, with an ∼2.5-fold increase in *K_m_*^app^ over the wild type. The leucine substitution at position 367 further helps in this move since the hydrogen bonds between the side chains of Arg266 and Glu367 are no longer present and α-14 is repelled from Arg266 due to the Leu side chain (Fig. 4C).

### Feedback inhibition.

We soaked both trehalose and T6P into apo OtsA crystals and solved structures with both ligands (Fig. 4D and E). In both structures, the OtsA active site assumes an open conformation and two compounds superpose almost perfectly at the active site, recapitulating all of the interactions for the donor glucose and also interacting with His199 and Arg286, the latter a residue that interacts with the phosphates of the donor substrate (Fig. 4D and E). The structure with T6P further shows the phosphate group of the product occupying a similar position as the phosphate group of G6P, interacting with the side chains of Tyr90, Gln146, Arg324, and Tyr145, a residue that does not interact with G6P (Fig. 4E). Interestingly, the buffer present under the crystallization condition, *N*-cyclohexyl-2-aminoethanesulfonic acid (CHES), is observed in both trehalose- and trehalose-6-phosphate-bound structures, occupying the site of the nucleotide donor with the sulfate group binding in a phosphate site and interacting with hydroxyl groups from both glucose units of trehalose (Fig. 4D and E).

After obtaining these structures, we expected to observe some degree of feedback inhibition of OtsA by trehalose given that, under physiological conditions, free trehalose is highly abundant inside mycobacterial cells, reaching up to 15% of the total organic material in actively growing Mycobacterium smegmatis cells (39), while T6P could not be detected in M. tuberculosis cell extracts (25). Trehalose was found to inhibit the enzyme with a 50% inhibitory concentration (IC_50_) of ∼24 mM (Fig. S5). These results suggest that under physiological conditions, OtsA can be regulated by trehalose, the final product of the pathway which is highly abundant inside mycobacterial cells (39).

10.1128/mBio.02272-19.5 FIG S5 Feedback inhibition by trehalose. Download FIG S5, DOCX file, 0.03 MB. Copyright © 2019 Mendes et al. 2019 Mendes et al. This content is distributed under the terms of the Creative Commons Attribution 4.0 International license.

### Identification of an allosteric site in *M. thermoresistibile* OtsA.

Trehalose is a multipurpose molecule that is abundant in mycobacteria. It not only can function as an energy reserve and as structural component of cell wall glycolipids but can also lead to the synthesis of glycogen through the TreS-Mak-GlgE pathway and be directly synthesized from glycogen from the TreX-TreY-TreZ pathway. It is therefore likely that trehalose synthesis through the OtsA-B pathway is under strong regulation.

**FIG 5:**
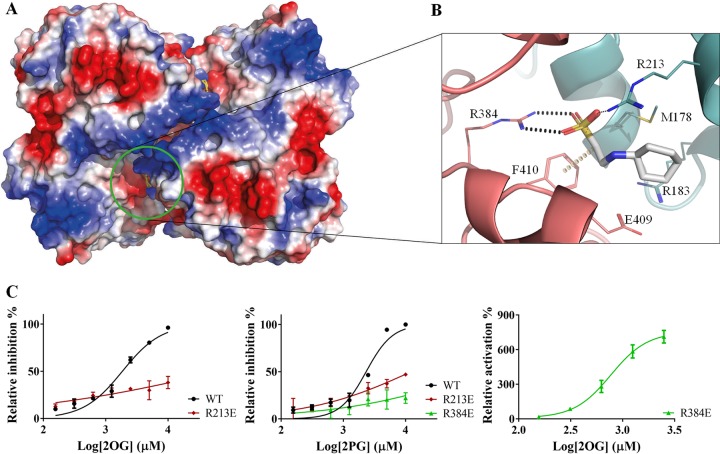
(A) View of the allosteric site of *M. thermoresistibile* OtsA with CHES bound with protein surface electrostatic potential shown. (B) Detailed view of the CHES binding site and its interactions with OtsA. The interactions were calculated with Arpeggio (61) using the apo structure (5JIJ). Black dots represent hydrogen bonds, and yellow disks represent a carbon-π interaction. The two protomers are colored differently. (C) Activity profiles of *M. thermoresistibile* wild-type OtsA and the allosteric site mutants Arg213Glu and Arg384Glu in the presence of the allosteric effectors 2OG and 2PG.

Under all the crystallization conditions that contained CHES, we could observe this compound in the crystal structures occupying a pocket formed by the contact interface of two OtsA protomers in the tetrameric assembly (Fig. 5). The CHES sulfate group directly interacts with the side chains of Arg384 of protomer A and Arg213 of protomer B, forming hydrogen bonds with both but also hydrogen-π interactions with Phe410 of protomer A (Fig. 5). The residues composing this site are completely conserved in mycobacterial and closely related species that are likely to harbor OtsA tetramers but not in other species known to have other oligomeric forms (Fig. S2).

Arg384 sits in a loop that forms extensive contacts with the donor substrate through Asp385, Gly386, Met387, and Leu389 (Fig. 2). Ligands interacting with the side chain of Arg384 could therefore have an impact on the activity of the enzyme, making this site a prime candidate for allosteric regulation.

While CHES had no discernible impact on OtsA activity at the tested concentrations, it was reported before that F6P acted as an allosteric regulator of M. tuberculosis OtsA (35), and the same was reported for the yeast OtsA-B complex (40). We have tested *Mtr*OtsA under the same conditions as those reported by Asencion Diez and colleagues (35) and could not detect any effect of F6P for *Mtr*OtsA. Although we did not observe any effect of F6P on enzyme activity for *Mtr*OtsAs, we nevertheless attempted to soak this compound in both apo form and OtsA:ADP:G6P ternary complex crystals and also cocrystallize it in the presence and absence of ADP and G6P, all under CHES-free conditions. None of these conditions provided a structure in which F6P occupied an allosteric site. We could, however, observe F6P when cocrystallized together with ADP, bound to the acceptor substrate site, with OtsA presenting the active site in a closed conformation (Fig. S6).

10.1128/mBio.02272-19.6 FIG S6 (A) View of the active site of *M. thermoresistibile* OtsA complexed with ADP (white) and F6P (yellow), with F6P occupying the acceptor site. (B) Fo-Fc “omit” map for the two ligands is shown contoured at 1.5 σ. Black dashed lines represent hydrogen bonds. Download FIG S6, DOCX file, 0.3 MB. Copyright © 2019 Mendes et al. 2019 Mendes et al. This content is distributed under the terms of the Creative Commons Attribution 4.0 International license.

OtsA was found to interact with a multitude of proteins in a large-scale proteomics study (41), including enolase, which catalyzes the penultimate step of glycolysis. In our search for an allosteric regulator, we therefore decided to test several glycolytic metabolites, glucose-1-phosphate (G1P), fructose-1,6-biphosphate (F16BP), 3-phosphoglycerate (3PG), the enolase substrate 2-phosphoglyceric acid (2PG), and the product phosphoenolpyruvate (PEP), but also the master metabolic regulators cAMP and 2-oxoglutarate (2OG) (42) and assess their impact in OtsA activity.

From the several compounds tested, only 2OG and 2PG showed clear inhibition of OtsA activity with IC_50_s of 1.8 mM and 2.3 mM, respectively (Table 2 and Fig. 5C). To assess whether they could be acting allosterically on *Mtr*OtsA and binding to the site identified by CHES, we performed two mutations on the arginines that interacted with CHES (Arg213Glu and Arg384Glu) and tested the two mutants’ activity in the presence of 2OG and 2PG. The results show that the Arg213Glu mutation abolished the strong response of the protein to 2OG while there was an ∼5-fold increase in 2PG IC_50_ to 12 mM. For the Arg384Glu mutant, 2OG unexpectedly becomes a strong activator with a 50% effective concentration (EC_50_) of 0.7 mM while 2PG has no effect (Table 2).

**TABLE 2:** Effect of allosteric regulators in *M. thermoresistibile* OtsA activity

Genotype	Value for allosteric regulator (95% confidence interval)			
	2OG		2PG	
	IC_50_ (mM)	*n*_H_^*b*^	IC_50_ (mM)	*n*_H_
Wild type	1.8 (1.6–2.1)	1.4 (1.1–1.7)	2.3 (2.0–2.7)	2.0 (1.4–2.7)
R213E	73 (18–302)	0.26 (0.16–0.35)	12 (6.3–24)	0.51 (0.40–0.63)
R384E	0.8 (0.6–0.9)^*a*^	2.5 (1.4–3.5)	<100	

a Value for 2OG for R384E is EC_50_ (mM), not IC_50_.

b *n*_H_, Hill coefficient.

## DISCUSSION

Trehalose, an essential disaccharide in mycobacteria, is one of the critical components of the cell wall. The OtsA-B pathway is an essential source of trehalose for these organisms, with knockouts of this pathway being growth defective or nonviable (24, 25). Here, we reveal the first mycobacterial OtsA structure. The overall structure of *M. thermoresistibile* OtsA is similar to the reported structures of E. coli, *S. venezuelae*, and Paraburkholderia xenovorans OtsAs despite relatively low sequence identity (30 to 35%). Remarkably, OtsAs also have a fold highly similar to the pseudoglycosyltransferases VldE and ValL involved in synthesis of validamycin A, a potent antifungal agent (43, 44).

Nevertheless, there are substantial differences in the oligomeric organization of bacterial OtsAs, with tetrameric, dimeric, and protomeric forms reported. While mycobacteria and related species present conservation of interfaces, showing that the observed tetrameric form for *Mtr*OtsA is present in all these organisms, beyond this group such conservation is absent, which is consistent with other oligomeric states (see Fig. S2 in the supplemental material).

The structures obtained in this work allow us to explain how mycobacterial OtsA prefers ADP-glucose as a substrate. A cavity found to accommodate the binding of the adenine moiety is surrounded with Leu319 and Arg361, which are critical for substrate recognition. It is further mediated by the Val363 carbonyl group, which does not allow GDP-glucose to occupy the same position, and by Glu367. Mutating these three residues to the E. coli OtsA equivalents completely changed the preference to UDP-glucose, the preferred E. coli OtsA donor substrate. Both Leu319 and Glu367 are highly conserved in all mycobacterial and closely related species analyzed but not beyond this group, while Val363 can be replaced by other small hydrophobic amino acids, such as alanine and isoleucine, in other mycobacterial species and closely related species (Fig. S2). However, outside this group Val363 is replaced by bulkier residues, such as tyrosine and phenylalanine (Fig. S2). These differences are suggestive of alternative donor substrate preferences for organisms outside the suborder *Corynebacterineae*.

Trehalose is not only abundant inside the mycobacterial cell but also involved in a cycle that links it to the synthesis and degradation of glycogen (45). We have shown that OtsA is feedback inhibited by trehalose but not by T6P, even though the structures obtained show that both compounds form extensive interactions with OtsA. However, given that these two structures were obtained by soaking apo-form crystals with high concentrations of trehalose and T6P and that CHES is also observed at the active site interacting with both compounds, it is possible that these two structures do not entirely reflect their natural interactions with the enzyme. The toxic accumulation of T6P in OtsB2-knockout mutants (25) can be explained by these results, since OtsA shows very low sensitivity to T6P. Furthermore, trehalose levels are reduced in the mutant (25), further contributing to an increase in OtsA activity, leading to higher T6P production.

Regulation of OtsA by phosphorylation and methylation has been observed for yeast trehalose-6-phosphate synthase complex (46, 47), and phosphorylation of E. coli OtsA has also been reported (48) in two residues close to the active site. Even though only one of the residues is conserved in mycobacteria (Ser323 for *Mtr*OtsA), phosphorylation of this residue would most likely lead to inactivation of the enzyme due to the location close to the acceptor binding site (Fig. S7). Interestingly, this residue is highly conserved in all sequences analyzed, with a single exception where it is replaced by a threonine (Fig. S2), suggesting that phosphorylation of this residue might be a common regulatory mechanism of OtsA activity.

10.1128/mBio.02272-19.7 FIG S7 View of the active site of *M. thermoresistibile* OtsA complexed with ADP and G6P (white). Distances between the Ser323 hydroxyl group and other atoms interacting with the phosphate group of G6P are shown. Phosphorylation of Ser323 would place the phosphate group in a position similar to the one occupied by G6P. Download FIG S7, DOCX file, 0.1 MB. Copyright © 2019 Mendes et al. 2019 Mendes et al. This content is distributed under the terms of the Creative Commons Attribution 4.0 International license.

Allosteric regulation was reported for both M. tuberculosis OtsA and yeast trehalose phosphate synthase complex, mediated by F6P (35, 40). The structure obtained with F6P shows this compound occupying the donor site, in the presence of ADP, with the enzyme adopting a closed conformation. Nevertheless, OtsA could use only G6P as an acceptor, and F6P showed no effect on the enzyme activity, indicating that the presence of F6P in the acceptor site was forced by the high concentration used for cocrystallization (5 mM). The observed differences in regulation by F6P between mycobacterial enzymes are difficult to reconcile since the allosteric site is highly conserved in mycobacterial OtsAs and closely related species (Fig. S2).

*Mtr*OtsA is allosterically regulated by 2PG, the substrate of enolase, a glycolytic enzyme, and 2OG, a master metabolic regulator that sits at the interface of carbon and nitrogen metabolism. The observed effect is within relevant physiological concentrations for both compounds (49, 50).

The effect of 2PG on OtsA and the reported interaction with enolase suggest an interplay between these two enzymes that would be interesting to explore further on the enolase side. Enolase is a multifunctional protein involved in a variety of cellular processes, beyond its enzymatic activity, which include response to oxidative and thermal stress (51). Given that trehalose is a known chemical chaperone and compatible solute (1), the association between enolase and OtsA, through the effect of 2PG on OtsA activity and the reported interaction between the two enzymes, points to further regulation of stress response.

2OG is a molecule that sits at the interface of carbon and nitrogen metabolism and that has been shown to regulate many different pathways (42). The role of trehalose as an energy reserve molecule (1) and its relationship with synthesis and degradation of glycogen (52) point to a mechanism in which 2OG influences the synthesis of these two compounds through regulation of OtsA activity, one that deserves to be further explored.

We have shown that OtsA can be allosterically inhibited; however, a single mutation at the allosteric site changed the behavior of 2OG, but not 2PG, from an inhibitor to an activator. This hints at the possible existence of allosteric activators of OtsA yet to be discovered.

The conservation of allosteric site residues and oligomeric assembly in the suborder *Corynebacterineae* but not outside suggests that allosteric regulation of OtsA through this site might be limited to this group of organisms. The results of this work are a significant step forward in understanding the regulation of trehalose synthesis in mycobacteria and the structural reasons behind substrate preference and provide new important insight into this enzyme.

## MATERIALS AND METHODS

### Bacterial strains and cloning.

The *M. thermoresistibile* (DSM 44167) *otsA* gene was amplified from chromosomal DNA obtained from the Deutsch Sammlung von Mikroorganismen und Zellkulturen GmbH (DSMZ; Braunschweig, Germany). Primers were designed based on the sequence available in the NCBI database, and the gene was cloned between the BamHI and HindIII sites in pET28a vector (Novagen), modified with an N-terminal 6×His-SUMO tag. The resulting plasmid was confirmed by DNA sequencing and transformed into E. coli strain BL21(DE3) (Invitrogen). Six *Mtr*OtsA mutants (Arg213Glu, Leu319Ile, Val363Phe, Arg384Glu, Leu319Ile-Glu367Leu, and Leu319Ile-Val363Phe-Glu367Leu) were also constructed by site-specific mutagenesis, sequenced, and transformed into E. coli strain BL21(DE3). Primers used in this work are listed in Table S3 in the supplemental material.

10.1128/mBio.02272-19.10 TABLE S3 Primers used in this work. Download Table S3, DOCX file, 0.01 MB. Copyright © 2019 Mendes et al. 2019 Mendes et al. This content is distributed under the terms of the Creative Commons Attribution 4.0 International license.

### Recombinant expression and protein purification.

Transformed E. coli BL21(DE3) cells were grown to mid-exponential growth phase (optical density at 610 nm [OD_610_] = 0.6) in LB medium (Invitrogen) containing 30 mg liter^−1^ kanamycin at 37°C. Isopropyl-β-d-1-thiogalactopyranoside (IPTG) was then added at a final concentration of 0.5 mM to induce gene expression, and the temperature was lowered to 18°C. Cells were harvested 18 to 20 h later by centrifugation and resuspended in 20 mM Tris (pH 7.5), 500 mM NaCl, and 20 mM imidazole with protease inhibitor tablets (Roche), DNase I, and 5 mM MgCl_2_. Cells were lysed by sonication, and cell lysate was centrifuged at 27,000 × *g* for 30 min to remove cell debris.

Recombinant *Mtr*OtsA was purified with a HiTrap IMAC Sepharose FF column (GE Healthcare), equilibrated with 20 mM Tris (pH 7.5), 500 mM NaCl, and 20 mM imidazole. Elution was performed in the same buffer but with 500 mM imidazole. Imidazole was removed with a desalting column, and SUMO tag was cleaved overnight at 4°C by adding Ulp1 protease at a 1:100 ratio in 20 mM Tris (pH 7.5), 500 mM NaCl. SUMO tag, Ulp1 protease, and uncleaved SUMO-OtsA were removed with a HiTrap IMAC Sepharose FF column (GE Healthcare), equilibrated with 20 mM Tris (pH 7.5), 500 mM NaCl, and 20 mM imidazole. Flowthrough containing OtsA was collected, concentrated, and loaded to a Superdex 200 column equilibrated with 20 mM Tris (pH 7.5), 500 mM NaCl. Fraction purity was determined by SDS-PAGE, and the purest fractions were pooled, concentrated to ∼10 mg · ml^−1^ in 20 mM Tris (pH 7.5)-350 mM NaCl, flash frozen in liquid nitrogen, and stored at −80°C. The same purification protocol was used for all *Mtr*OtsA mutants.

### Crystallization and data collection.

*Mtr*OtsA crystallization screens and optimization were performed at 18°C using the sitting-drop vapor diffusion method. Three hundred nanoliters of pure OtsA at 10 mg · ml^−1^ was mixed in a 1:1 ratio with well solution using a Phoenix robot (Art Robbins). Initial conditions were obtained in the Classics Suite crystallization screen (Qiagen), solution 36. Crystals obtained under this condition diffracted only up to 3 Å. Therefore, further optimization was performed using the additive screen HT (Hampton Research), and ethylene glycol was found to be the best additive. The final optimized condition consisted of 0.7 M sodium potassium tartrate, 0.1 M CHES (pH 10), and 10% (vol/vol) ethylene glycol. Crystals appeared after 4 days under this condition. To obtain ligand-bound structures, soaking was performed under the optimized condition using the hanging-drop vapor diffusion method as follows: 1 μl of protein storage buffer containing 5 mM ligand was mixed with 1 μl of reservoir solution, and drops were left to equilibrate against 500 μl of reservoir solution for 3 days. Crystals were then transferred to the preequilibrated drops and incubated for 24 h. A cryogenic solution was prepared by adding ethylene glycol up to 25% (vol/vol) to mother liquor. Crystals were briefly transferred to this solution, flash frozen in liquid nitrogen, and stored for data collection. To obtain an ADP-G6P-OtsA ternary complex, cocrystallization with 5 mM ADP and G6P was performed instead, as all attempts to soak G6P alone or G6P in the presence of ADP have failed. Crystals were obtained in the Wizard Classic I&II screen (Rigaku), solution E10, and were flash frozen in liquid nitrogen after a brief soak in a solution containing mother liquor and 25% ethylene glycol. The same condition was used to obtain ADP:F6P:OtsA complex.

All data sets were collected at stations I04, I04-1, and I24 at Diamond Light Source (Oxford, United Kingdom). Data collection and refinement statistics are summarized in Table S1.

### Structure solution and refinement.

Diffraction data were processed and reduced using MOSFLM (53) and Aimless (54) from the CCP4 suite (55) or autoPROC from Global Phasing Limited (56). The apo form crystalized in the I4_1_22 space group with one protomer per asymmetric unit. The ADP-G6P-OtsA ternary complex crystallized in the P6_2_22 space group, again with one protomer per asymmetric unit.

Initial phases were determined with Phaser (57) from the PHENIX software package (58) using the structure of E. coli OtsA (PDB entry 1UQU) (32) as a search model. Model building was done with Coot (59), and refinement was performed in PHENIX (58). Structure validation was performed using Coot and PHENIX tools (58, 59). All the figures were prepared with PyMOL (http://www.pymol.org).

### Multiple sequence alignment.

The multiple sequence alignment was performed with T-Coffee (60) using the OtsA sequences of Mycobacterium thermoresistibile, Mycobacterium tuberculosis, Mycobacterium leprae, Mycobacterium avium, Mycobacterium abscessus, Nocardia farcinica, Arthrobacter alpinus, Salmonella enterica serovar Typhimurium, Escherichia coli, Candida albicans, Paraburkholderia xenovorans, and Streptomyces venezuelae.

### Prediction of mutations in ligand affinity.

To provide an insight on which residues to mutate, we used mCSM-lig, a software program that predicts the effect of mutations in ligand affinity (38) on the X-ray crystal structure of OtsA with ADP-glucose (5JIO).

### Enzymatic assays.

Formation of T6P was assessed by a continuous colorimetric assay that followed the release of NDP by measuring the oxidation of NADH at 340 nm, in the presence of pyruvate kinase and lactate dehydrogenase. All reagents used were obtained from Sigma-Aldrich. The enzymatic reactions (200-μl reaction mixture) were performed at 37°C, and the reaction mixtures contained 50 mM Tris (pH 7.5), 200 mM NaCl, 10 mM MgCl_2_, 50 mM KCl, 0.3 mM NADH, 2.5 mM phosphoenolpyruvate (PEP), 10 units of pyruvate kinase-lactate dehydrogenase, 0.1 μM enzyme, and various concentrations of G6P and NDP-glucose to determine kinetic parameters.

To assess the specificity of OtsA for G6P, several compounds (F6P, mannose-6-phosphate, glucosamine-6-phosphate, and G1P) were tested for their capacity to act as acceptor substrates, under the conditions above, using ADP-glucose as the donor substrate. The effect of trehalose and T6P as feedback inhibitors and of G1P, F6P, F16BP, CHES, 2PG, 3PG, cAMP, and 2OG as allosteric regulators was tested under the conditions described above but with fixed concentrations of G6P and ADP-glucose (both 0.3 mM). The same conditions were used to test possible allosteric regulators for wild-type OtsA and mutants Arg213Glu and Arg384Glu. All experiments were performed in triplicate in a PHERAstar plate reader (BMG Labtech), and data were analyzed with Prism 5 (GraphPad Software).

To assess the inhibitory effect of ADP and the allosteric effect of phosphoenolpyruvate, an endpoint assay was performed instead. Reaction mixtures (100 μl) containing 50 mM Tris (pH 7.5), 200 mM NaCl, 10 mM MgCl_2_, 50 mM KCl, 4 μM *Mtr*OtsA, 0.3 mM ADP-glucose, 0.3 mM G6P, and various concentrations of ADP and PEP were incubated at 37°C; the reactions were stopped at different time points with 10 μl of 1 M HCl; and the reaction mixtures were incubated for 1 min and neutralized with NaOH. The reaction mixtures were diluted with 100 μl acetonitrile, and the reactions were run at 25°C on a Waters I-class ultrahigh-performance liquid chromatograph (UHPLC) with a photodiode array (PDA) detector (258 nm) using an Acquity UHPLC ethylene-bridged hybrid (BEH) amide column (2.1 by 100 mm, 1.7 μm). Gradient elution (delivered at 0.13 ml/min) was employed using 80/20 acrylamide-water with 0.1% NH_2_OH (A) and water with 0.1% NH_2_OH (B), which started at 90% A and decreased linearly to 55% A over 10 min.

### Isothermal titration calorimetry.

Binding interaction between OtsA and ligands was characterized at 25°C, using a MicroCal ITC200 titration calorimeter (MicroCal). An OtsA concentration of 60 μM was used for all titrations. Ligands (1 mM) were injected in 2-μl aliquots. Titration data were recorded in 20 mM Tris (pH 7.5)-500 mM NaCl. Data were analyzed by fitting a simple single-site model using Origin software (MicroCal).

### Accession numbers.

The coordinates and structure factors presented in this work have been deposited in the Protein Data Bank under accession codes 5JIJ, 5K41, 5K42, 5JIO, 5L3K, 5K5C, and 5K44.
