# Supplementary material for: Mycobacterial OtsA Structures Unveil Substrate Preference Mechanism and Allosteric Regulation by 2-Oxoglutarate and 2-Phosphoglycerate
Source: mBio. 2019 Nov 26;10(6):e02272-19. doi: 10.1128/mBio.02272-19 (PMC6879718; doi:10.1128/mBio.02272-19)
Supplement: FIG S1 [file mBio.02272-19-sf001.docx]

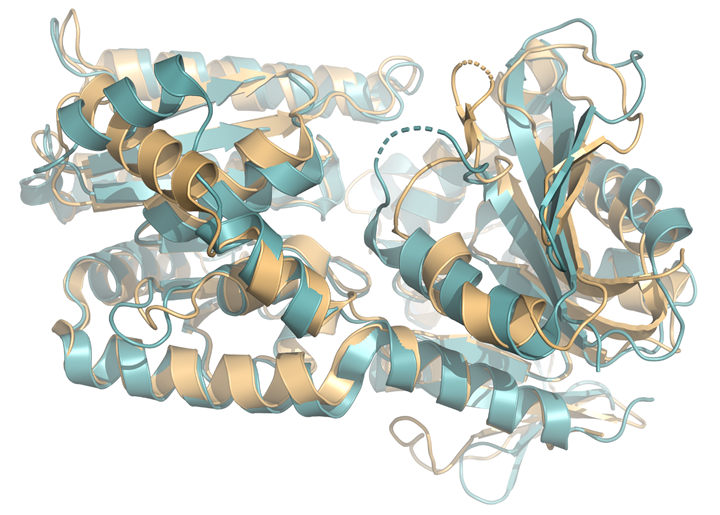


**Figure S1**: Alignment of the X-ray crystal structures of *E. coli* OtsA (PDB code: 1UQU) in cyan and *M. thermoresistibile* OtsA Apo form in orange (RMSD = 2.996).
