# Supplementary material for: Mycobacterial OtsA Structures Unveil Substrate Preference Mechanism and Allosteric Regulation by 2-Oxoglutarate and 2-Phosphoglycerate
Source: mBio. 2019 Nov 26;10(6):e02272-19. doi: 10.1128/mBio.02272-19 (PMC6879718; doi:10.1128/mBio.02272-19)
Supplement: TABLE S1 [file mBio.02272-19-st001.docx]

**Table S1:** X-ray crystallography data collection and final refinement statistics

| Ligand# | **APO** | **ADP-glucose** | **GDP-glucose** | **ADP G6P** | **ADP-F6P** | **Trehalose** | **T6P** |
| --- | --- | --- | --- | --- | --- | --- | --- |
| PDB ID | 5JIJ | 5K41 | 5K42 | 5JIO | 5L3K | 5K5C | 5K44 |
| **Data collection*** |  |  |  |  |  |  |  |
| Space group | *I*4_1_22 | *I*4_1_22 | *I*4_1_22 | *P*6_2_22 | *P*6_2_ | *I*4_1_22 | *I*4_1_22 |
| Cell parameters:  a [Å]  b [Å]  c [Å]  α/β/γ [˚] | 126.86  126.86  207.19  90/90/90 | 127.41  127.41  205.62  90/90/90 | 127.31  127.31  206.78  90/90/90 | 105.36  105.36  158.87  90/90/120 | 216.96  216.96  159.84  90/90/120 | 127.32  127.32  207.38  90/90/90 | 127.23  127.23  207.61  90/90/90 |
| Resolution range [Å] | 60.66 – 1.82  (1.86 – 1.82) | 102.81 – 1.97  (2.20 – 1.97) | 108.41 – 1.92  (2.15 – 1.92) | 91.24– 1.71  (1.87 – 1.71) | 159.84 – 2.31  (2.43 – 2.31) | 103.69 – 1.85  (2.02 – 1.85) | 103.80 – 1.93  (2.15 – 1.93) |
| No. of observations  total  unique | 833006  (50109)  75576  (4427) | 753000  (216413)  59763  (16773) | 842990  (231847)  64773  (18176) | 1030194  (193800)  56816  (13323) | 1927301  (266711)  187701  (27326) | 923936  (184758)  72774  (17204) | 817022  (229455)  64496  (18114) |
| R_merge_ | 0.116 (0.716) | 0.154 (0.669) | 0.078 (0.958) | 0.096 (0.790) | 0.268 (1.138) | 0.076 (0.762) | 0.093 (0.544) |
| I/σ(I) | 9.5 (2.0) | 9.5 (2.8) | 23.1 (3.3) | 19.1 (3.3) | 7.0 (2.1) | 20.1 (3.0) | 15.6 (3.5) |
| Completeness [%] | 100.0 (100.0) | 100.0 (99.9) | 100.0 (99.9) | 100.0 (100.0) | 99.9 (99.9) | 100.0 (100.0) | 100.0 (100.0) |
| Multiplicity | 11.0 (11.3) | 12.6 (12.9) | 13.0 (12.8) | 18.1 (14.5) | 10.3 (9.8) | 12.7 (10.7) | 12.7 (12.7) |
| **Refinement** |  |  |  |  |  |  |  |
| Refinement program | PHENIX | PHENIX | PHENIX | PHENIX | PHENIX | PHENIX | PHENIX |
| Resolution [Å] | 60.66 – 1.82 | 90.09 – 1.97 | 45.01 – 1.92 | 91.24 – 1.71 | 121.74 – 2.31 | 41.58 – 1.85 | 40.23 – 1.93 |
| No. reflections | 74139 | 59634 | 64577 | 56756 | 187654 | 72668 | 64406 |
| R_work_/R_free_ [%] | 16.5/18.6 | 16.5/19.1 | 15.6/18.4 | 15.2/17.9 | 15.3/21.3 | 15.1/17.5 | 14.9/17.4 |
| RMS deviations |  |  |  |  |  |  |  |
| Bonds [Å] | 0.007 | 0.007 | 0.007 | 0.007 | 0.009 | 0.007 | 0.008 |
| Angles [˚] | 1.009 | 1.042 | 1.045 | 1.092 | 1.048 | 1.028 | 1.030 |
| Ramachandran |  |  |  |  |  |  |  |
| Favoured [%] | 97 | 97 | 97 | 97 | 95 | 97 | 96 |
| Outliers [%] | 0 | 0 | 0 | 0 | 0 | 0 | 0 |
| Average B-factor [Å^2^] |  |  |  |  |  |  |  |
| macromolecule | 40.3 | 44.8 | 44.9 | 31.2 | 32.6 | 40.2 | 40.7 |
| ligands | 49.3 | 43.0 | 66.0 | 25.8 | 30.9 | 45.5 | 43.0 |
| solvent | 43.7 | 49.2 | 50.0 | 39.8 | 30.8 | 47.6 | 48.0 |

* Parameters shown in brackets are for the highest resolution shell
