# Supplementary material for: Mycobacterial OtsA Structures Unveil Substrate Preference Mechanism and Allosteric Regulation by 2-Oxoglutarate and 2-Phosphoglycerate
Source: mBio. 2019 Nov 26;10(6):e02272-19. doi: 10.1128/mBio.02272-19 (PMC6879718; doi:10.1128/mBio.02272-19)
Supplement: FIG S2 [file mBio.02272-19-sf002.docx]

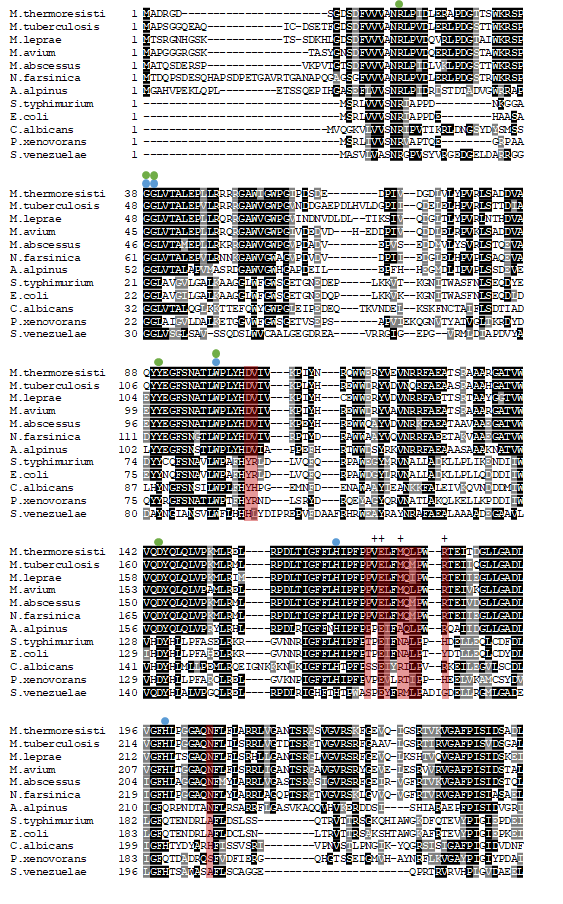


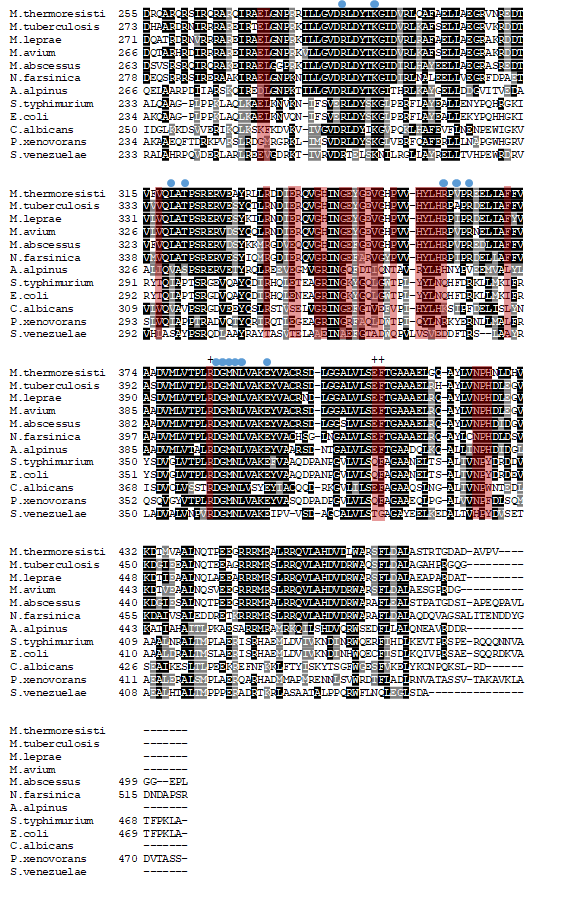


**Figure S2:** Sequence comparison of OtsA from *Mycobacterium thermoresistibile*, *Mycobacterium tuberculosis*, *Mycobacterium leprae*, *Mycobacterium avium*, *Mycobacterium abscessus*, *Nocardia farcinica*, *Arthrobacter alpinus*, *Salmonella typhimurium*, *Escherichia coli*, *Candida albicans*, *Paraburkholderia xenovorans* and *Streptomyces venezuelae*. Residues that contact with the substrates are highlighted with blue circles (donor site) and green circles (acceptor site). The allosteric site residues are marked with crosses and tetramer interfaces are highlight in red. The tetramer interfaces are highly conserved only in the several mycobacteria and *N. farcinica* and less *A. alpinus*. The remaining non-actinobacterial species show very little conservation of the interfaces. The same is observed for the allosteric site. Acceptor site residues are conserved throughout. Donor site residues are less conserved indicating the known differences in substrate preference.
