# Supplementary material for: Mycobacterial OtsA Structures Unveil Substrate Preference Mechanism and Allosteric Regulation by 2-Oxoglutarate and 2-Phosphoglycerate
Source: mBio. 2019 Nov 26;10(6):e02272-19. doi: 10.1128/mBio.02272-19 (PMC6879718; doi:10.1128/mBio.02272-19)
Supplement: FIG S3 [file mBio.02272-19-sf003.docx]

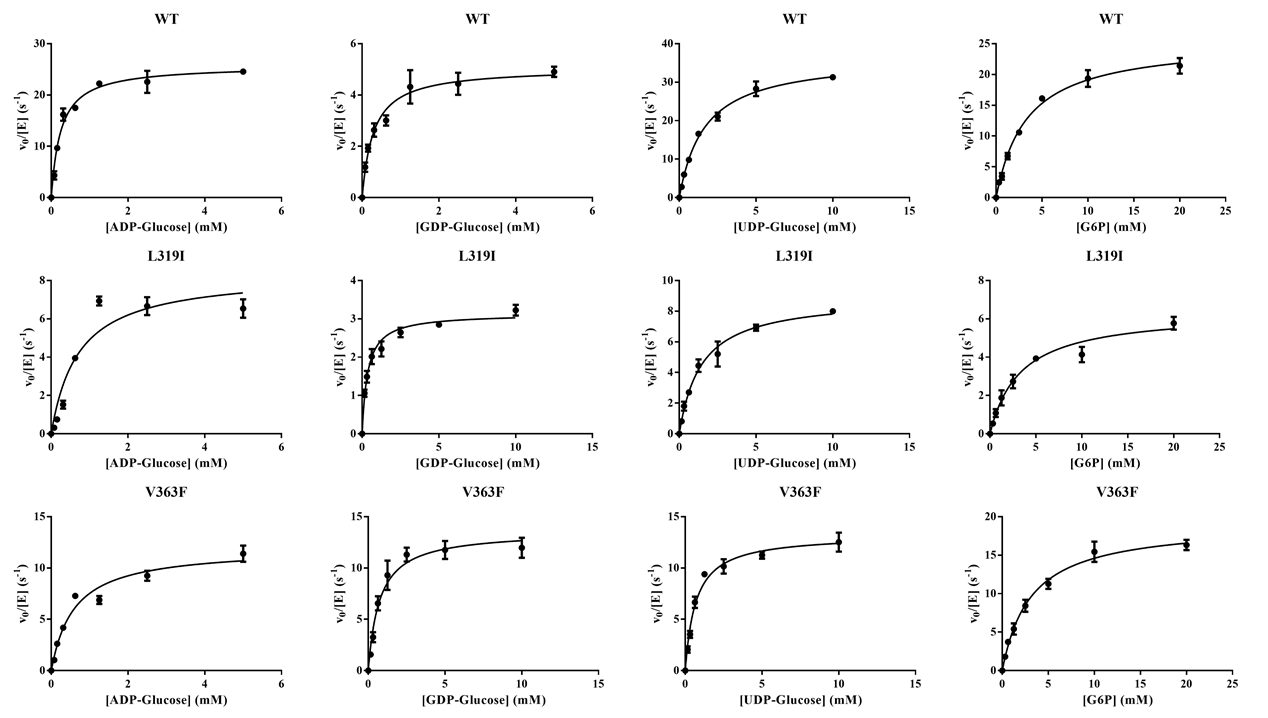


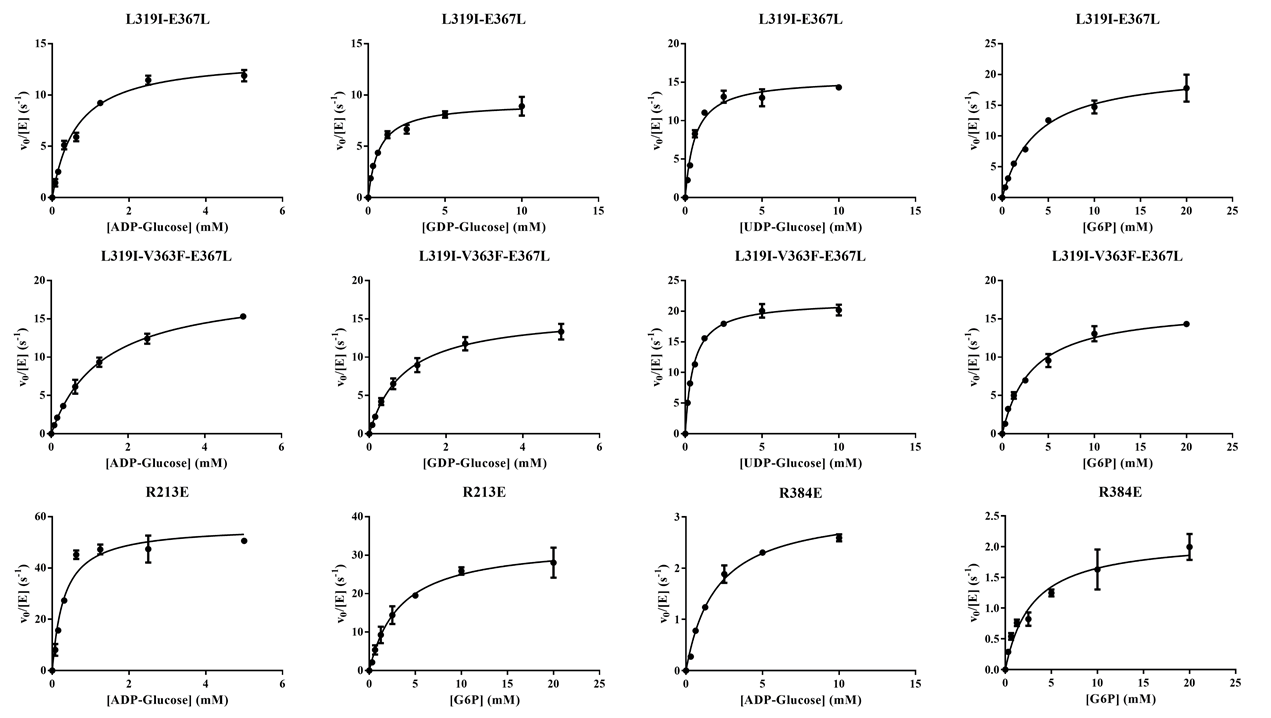


**Figure S3:** *Mtr*OtsA wt and mutants kinetics. G6P concentration was fixed at 10 mM for all NDP-glucose. ADP-glucose concentration was kept at 2.5 mM for G6P.
