# Supplementary figures and images for: Mycobacterial OtsA Structures Unveil Substrate Preference Mechanism and Allosteric Regulation by 2-Oxoglutarate and 2-Phosphoglycerate
Source: mBio. 2019 Nov 26;10(6):e02272-19. doi: 10.1128/mBio.02272-19 (PMC6879718; doi:10.1128/mBio.02272-19)

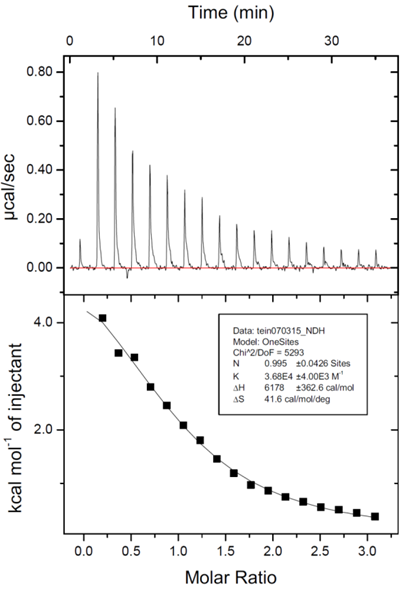


**Figure S4:** ITC trace with *Mtr*OtsA for ADP-glucose binding

Supplement: FIG S4 [file mBio.02272-19-sf004.docx]

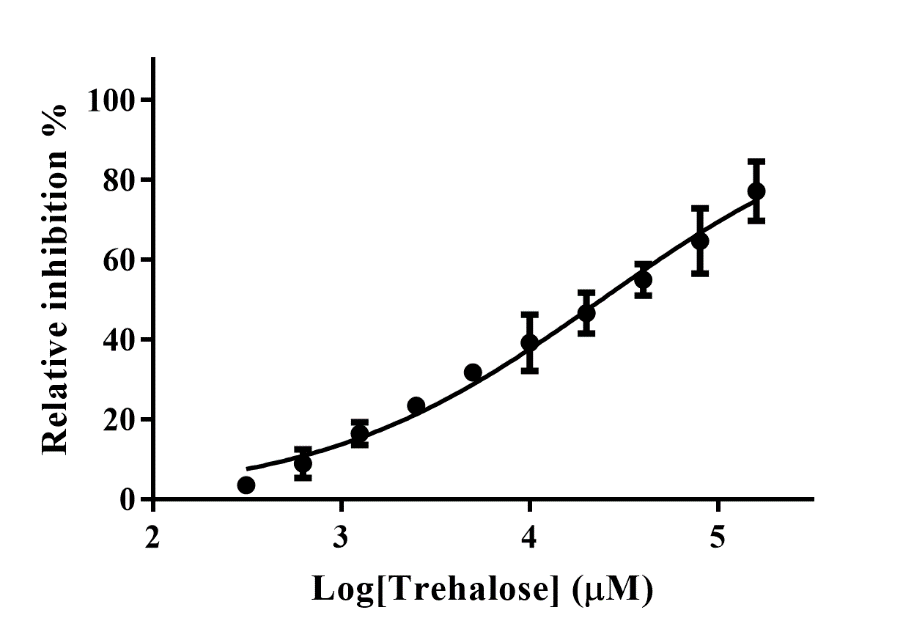


**Figure S5:** Feedback inhibition by trehalose.

Supplement: FIG S5 [file mBio.02272-19-sf005.docx]
