# Supplementary material for: Mycobacterial OtsA Structures Unveil Substrate Preference Mechanism and Allosteric Regulation by 2-Oxoglutarate and 2-Phosphoglycerate
Source: mBio. 2019 Nov 26;10(6):e02272-19. doi: 10.1128/mBio.02272-19 (PMC6879718; doi:10.1128/mBio.02272-19)
Supplement: TABLE S2 [file mBio.02272-19-st002.docx]

**Table S2:** mCSM-lig predicitons

| Residues | Log change in affinity |
| --- | --- |
| *V363F | -1.4 |
| D285E | -1.3 |
| T321P | -1.3 |
| *L319I | -1.1 |
| *E367L | -0.8 |
| P363H | -0.6 |
|  |  |

*Residues selected for mutation. All residues except E367 are within 4.5 Å of the adenine moiety of ADP-glucose.
