# Supplementary material for: Mycobacterial OtsA Structures Unveil Substrate Preference Mechanism and Allosteric Regulation by 2-Oxoglutarate and 2-Phosphoglycerate
Source: mBio. 2019 Nov 26;10(6):e02272-19. doi: 10.1128/mBio.02272-19 (PMC6879718; doi:10.1128/mBio.02272-19)
Supplement: FIG S6 [file mBio.02272-19-sf006.docx]

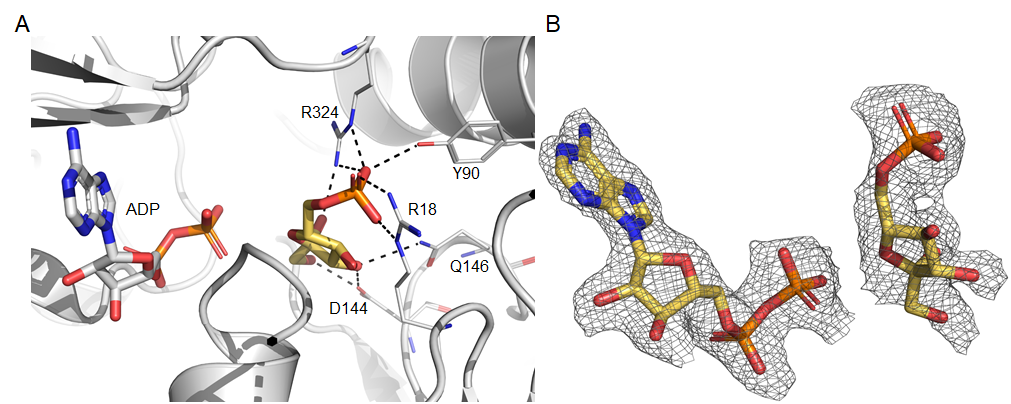


**Figure S6:** (A) View of the active site of *M. thermoresistibile* OtsA complexed with ADP (white) and F6P (yellow), with F6P occupying the acceptor site. Fo-Fc “Omit” map for the two ligands is shown contoured at 1.5 σ (B). Black dashed lines represent hydrogen bonds.
