# Supplementary material for: Mycobacterial OtsA Structures Unveil Substrate Preference Mechanism and Allosteric Regulation by 2-Oxoglutarate and 2-Phosphoglycerate
Source: mBio. 2019 Nov 26;10(6):e02272-19. doi: 10.1128/mBio.02272-19 (PMC6879718; doi:10.1128/mBio.02272-19)
Supplement: FIG S7 [file mBio.02272-19-sf007.docx]

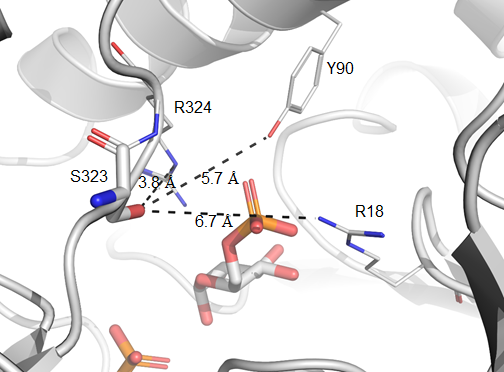


**Figure S7:** View of the active site of *M. thermoresistibile* OtsA complexed with ADP and G6P (white). Distances between Ser323 hydroxyl group and other atoms interacting with the phosphate group of G6P are shown. Phosphorylation of Ser323 would place the phosphate group in a similar position to the one occupied by G6P.
