# Supplementary material for: Mycobacterial OtsA Structures Unveil Substrate Preference Mechanism and Allosteric Regulation by 2-Oxoglutarate and 2-Phosphoglycerate
Source: mBio. 2019 Nov 26;10(6):e02272-19. doi: 10.1128/mBio.02272-19 (PMC6879718; doi:10.1128/mBio.02272-19)
Supplement: TABLE S3 [file mBio.02272-19-st003.docx]

**Table S3:** Primers used in this work

| *otsA*pET28SUMO_F | ATAGGATCCATGGCTGACCGGGGCGACTC |
| --- | --- |
| *otsA*pET28SUMO_R | ATTAAGCTTTCACACCGGAACCGCGTCGG |
| L319I_F | GACACCGTCTTCGTCCAGATCGCCACCCCCAGCCGCGAG |
| L319I_R | CTCGCGGCTGGGGGTGGCGATCTGGACGAAGACGGTGTC |
| V363F _F | CCTGCACCGGCCGTTTCCGCGTGAGGAAC |
| V363F _R | GTTCCTCACGCGGAAACGGCCGGTGCAGG |
| V363F_E367L_F | CCTGCACCGGCCGTTTCCGCGTGAGCTAC |
| V363F_E367L_R | GTAGCTCACGCGGAAACGGCCGGTGCAGG |
| R213E_F | CTTCCTGTTCCTGGCGCGAGAGCTGGTGGGCGCCAACAC |
| R213E_R | GTGTTGGCGCCCACCAGCTCTCGCGCCAGGAACAGGAAG |
| R384E_F | CATGCTGGTCACCCCGCTGGAGGACGGGATGAACCTGGT |
| R384E_R | ACCAGGTTCATCCCGTCCTCCAGCGGGGTGACCAGCATG |
